# The complete protections induced by the oil emulsion vaccines of the novel variant infectious bursal disease viruses against the homologous challenges indicating the important roles of both VP2 and VP1 in the antigenicity and pathogenicity of the virus

**DOI:** 10.3389/fvets.2024.1466099

**Published:** 2024-08-29

**Authors:** Weiwei Wang, Yu Huang, Yan Zhang, Yuanzheng Qiao, Jun Shi, Jianni Huang, Teng Huang, Tianchao Wei, Meilan Mo, Xiumiao He, Ping Wei

**Affiliations:** ^1^Institute for Poultry Science and Health, Guangxi University, Nanning, China; ^2^Guangxi Key Laboratory for Polysaccharide Materials and Modifications, School of Marine Sciences and Biotechnology, Guangxi Minzu University, Nanning, China

**Keywords:** novel variant infectious bursal disease virus (nvIBDV), oil emulsion inactivated vaccine (OEV), booster immunization, immune protection, antigenicity and pathogenicity

## Abstract

Novel variant infectious bursal disease virus (nvIBDV) is an emerging genotype (A2dB1b) that can cause severe and prolonged immunosuppression in young chickens. Despite current commercial vaccines being proven to lack complete protection against nvIBDV, it remains unclear whether the oil emulsion inactivated vaccines (OEVs) of the homologous and heterologous virus or booster immunization can provide effective protection. In this study, OEVs with two types of nvIBDV isolates QZ191002 (A-nv/B-nv) and YL160304 (A-nv/B-HLJ0504-like) were prepared and evaluated the protective effects of OEVs plus the booster immunizations with different current commercial vaccines against the challenge of nvIBDVs. The results from vaccination-challenge experiments showed that nvIBDV could break through the protection provided by only one immunization dose of the commercial vaccines, with the protection rates ranging from 40% to 60%. Interestingly, even with booster immunization with different commercial vaccines, the protection rates could only be increased to 60%–80%. As expected, only the OEVs of the homologous virus could provide 100% protection against the homologous nvIBDV, which could induce high-level specific antibodies, ameliorate target organ damage, and significantly reduce the viral load of the bursal in the challenged chickens. Notably, YL160304-OEV performed better than QZ191002-OEV, providing 100% protection not only against the challenge of homologous strain but also against that of heterologous QZ191002 strain. Antibody levels of the immunized chickens gradually increased after a short decline and reached the highest level on the age of 28 days. Similarly, the percentages of lymphocytes CD4+, CD8+ T, and B in peripheral blood lymphocytes (PBLs) were significantly increased on 21 d and 28 d. Notably, despite the nvIBDV, OEVs initially induced a delayed responses in the early stages but ultimately reach higher levels of CD4+ and CD8+ T lymphocytes. The results of study suggest that even booster immunization with different commercial vaccines cannot provide complete protection against nvIBDV, while the OEVs made by the nvIBDVs can provide full protection. Moreover, YL160304-OEV exhibits a broader protective spectrum against different nvIBDV strains, making it a potential candidate for the development of new vaccine.

## Highlights

- This study finds that even the booster immunization with the current commercial vaccines still cannot provide complete protection against the nvIBDV according to the current vaccination procedure.
- The oil emulsion vaccine (OEV) made by two inactivated nvIBDV isolates (QZ191002 and YL160304) can induce high-level specific antibodies, ameliorate target organ damage, and significantly reduce the viral load of the challenged virus in the vaccinated chickens and provide complete protection against the nvIBDV.
- The enhanced protective spectrum of the OEV of YL160304 (A-nv/B-HLJ0504-like vvIBDV) compared to QZ191002 (A-nv/B-nv) highlights that the role of VP1 is not only involved in the virulence of the virus but also seems to have a certain impact on the antigenicity of the virus and indicated that YL160304 strain could be a potential candidate for the development of new vaccine against the nvIBDV.

## 1 Introduction

Infectious bursal disease (IBD), caused by infectious bursal disease virus (IBDV), is a worldwide epidemic immunosuppressive disease in young chickens that have lymphoid tissue, especially the bursa of Fabricius (BF) of the central immune organ as its primary target (1, 2). IBDV, a member of the family of *Birnaviridae*, is a non-enveloped double-stranded RNA virus with high resistance to harsh environmental conditions. The IBDV genome consists of two segments (A and B) (3). Segment A encodes VP5 protein and, in another and partially overlapping reading frame, a polyprotein that will yield outer capsid protein VP2, protease VP4, and scaffold protein VP3 upon co-translational cleavage by VP4 (4), whereas segment B encodes VP1, the viral RNA-dependent RNA polymerase (RdRp), which exists in the viral capsids both as a genome-linked and as a free protein (5). IBDV specifically infects and lyses B lymphocytes, resulting in lymphoid depletion of B cells and atrophy of the bursa, which culminates in severe and long-term immunosuppression (6). The immunosuppressive effect of the disease reduces the bird’s resistance to other infections and reduces its immune responsiveness to the vaccinations and nature infections.

Two serotypes of the virus (serotypes 1 and 2) have been described, and only serotype 1 virus is pathogenic to chickens (7). Serotype 1 virus can be further divided into several pathogenic types, including classical (cIBDV) (8), very virulent (vvIBDV) (9), and antigenic variant (avIBDV) strains (10). The infections of different pathogenic IBDVs in chickens resulted in different clinical symptoms and pathogenic changes. cIBDV infection is characterized by bursal inflammation, severe lymphocyte necrosis, and immune damage, resulting in a mortality rate of 20%–30% in chickens (11, 12). attIBDV, which does not cause obvious clinical symptoms, is usually a domesticated attenuated vaccine strain that can protect against homologous wild IBDV strains. The characteristic of vvIBDV infection is that it can break through the protection of maternal antibodies, with mortality rate as high as 30%–100% (13, 14). In contrast, avIBDV does not lead to high mortality, often manifesting as a subclinical infection. Especially, the Chinese novel variant virus (nvIBDV) does not cause clinical morbidity and death, but it can cause serious damage to the BF and have a certain impact on the weight gain of chickens (15–24). Notably, compared to vvIBDV, nvIBDV exhibits significant differences in both VP2 and VP1, with a total of nine amino acid (aa) mutations (N77D, D213N, Q221K, A222T, Q249K, G254N, T289I, G318D, and D323E) in the antigen-associated projection (P) domain of VP2, seven mutations in the N-terminal domain (V4I, A24V, I61V, V/L141I, T145N, N/G147D, and A163V), five mutations in the central polymerase domain (D240E, A287T, S511R, E515D, and S646G), and one mutation in the C-terminal domain (P687S) of VP1 (20). Most of these mutations on VP2 and VP1 are related to the cell tropism, antigenic variation, and virulence diversity among IBDV strains (25–33). This may be an important factor (significant differences in the protective antigen-associated genes) why current commercial vaccines (mainly targeting the epidemic vvIBDV strains) cannot provide complete protection against nvIBDV.

In addition to strict biosafety measures, massive immunization is the optimal strategy and effective method to prevent and control IBDV infection. The immune effect of vaccines is closely related to effectively stimulating the immune defense system of chickens (34, 35). At present, commercial IBD vaccines mainly include live-attenuated, immune complex, and whole-virus inactivated vaccines (36, 37). Live-attenuated IBD vaccines can replicate in the bursa and produce humoral and cellular immunity simultaneously (38). However, large-scale use of these vaccines in the poultry industry may cause reassortment and recombination risks with field strains, thereby accelerating the variation of IBDV (20, 39, 40). In addition, there is a risk of them returning to a virulent form and causing immunosuppression, resulting in diseases in normal or immune-impaired chickens (41). Furthermore, these vaccines contain intact pathogens, making it difficult to distinguish infected chickens from immunized chickens in disease management. Compared with live-attenuated IBD vaccines, inactivated vaccines are considered to be safer and more effective in stimulating and inducing IBDV-specific antibodies and T-cell immune responses (36, 42), but they cannot establish long-term immunity (often requiring repeated immunization) (36). Therefore, these vaccines rely on adjuvants that stimulate strong and long-term humoral and cellular immune responses, which are essential for the prevention of infectious viruses. Oil emulsion adjuvant, also known as water-in-oil emulsion, can retain the antigen at the injection site, leading to the continuous release of the antigen, and then inducing long-term efficacy in poultry (43), which is one of the most common and effective adjuvants used currently in the veterinary field.

Some research groups have demonstrated that current commercial vaccines cannot provide complete protection against the increasing challenge of nvIBDV infection (20, 22, 44, 45). However, these research studies were only based on one immunization dose, which is different from the twice immunization dose used in the current immunization practice. This raises the possibility that incomplete protection might be due to an insufficient immune response stimulated and induced by once immunization dose. Therefore, we adopted the twice immunization regimen, following current production practices, and explored whether booster immunization with different vaccines can enhance immune responses to better resist the challenges posed by nvIBDV. Concurrently, we assessed the immunization efficacy of OEVs prepared from homologous nvIBDV strains. In this study, we developed two safe and effective oil emulsion inactivated vaccines (OEVs) based on the recently circulating nvIBDV isolates (20) and evaluated the protective effects of the newly developed nvIBDV OEVs and the booster immunization with different commercial vaccines according to the current clinical immunization practice procedures for the first time.

## 2 Materials and methods

### 2.1 Viruses and the commercial vaccines

Two purified nvIBDV strains QZ191002 (genotype A2dB1b, A-nv/B-nv) and YL160304 (genotype A2dB3, A-nv/B-HLJ0504-like vvIBDV), whose segment A derived from the same origin while segment B derived from different origin, were isolated and identified in the natural outbreaks of IBD that involved previously immunized chickens in southern China (20). The commercial vaccines A (IBD VP2 subunit vaccine), B (IBD immunogenic complex vaccine), C (IBD intermediate-plus live vaccine), and B87 attenuated vaccine used in this study were purchased from the commercial vaccine market.

### 2.2 Virus propagation and titer determination

As mentioned earlier, two types of nvIBDV strains propagate and titrate on HD11 cells (chicken macrophages cell line) that has been proven to be suitable for nvIBDV proliferation (20, 46, 47). In short, the virus was taken out from the preservation solution in the −80°C refrigerator, and the viruses were inoculated onto monolayer cells of 80%–85% confluent and cultured at 37°C for 1–2 h for virus adsorption, respectively. Subsequently, the inoculum was discarded and replaced with Dulbecco’s Modified Eagle Medium (DMEM) containing 1% fetal bovine serum (Lonsera, Shanghai Shuangru Biology Science & Technology Co., Ltd.) incubated for 72 h. When the cytopathic effect (CPE) was over 80%, cell cultures were harvested and then freeze-thawed three times. The harvested suspension was used to titrate the virus concentration according to the method previously described (48).

### 2.3 Virus inactivation and the preparation of the OEVs

The viruses were continuously inactivated with a final concentration of 0.4% formaldehyde in a shaking incubator at 37°C for 24 h. After the inactivated virus was determined to be sterile by sterility test, the inactivation efficiency was checked by three passages of each inactivated virus in specific pathogen-free (SPF) chicken embryos via the chorioallantoic membrane (CAM) route. The OEVs of the QZ191002 and YL160304 strains were prepared as the previously described method (49). In brief, add 4 parts of the sterilized Tween 80 to 96 parts of the inactivated virus was used as the aqueous phase. The oil phase, consisted of 90 parts No. 10 mineral oil and 10 parts Span 80, was sterilized by autoclaving. The aqueous and oil phases are emulsified in a ratio of 40/60 (v/v) at high speed (6000–10,000 r/min) and stored at 4°C until use.

### 2.4 Chicken vaccination-challenge experiment

A total of 230 1-day-old chicks of three-yellow chicken were purchased in a local commercial hatchery. All these chicks without any prior immunization were randomly divided into 23 groups (n = 10/group) with unrestricted access to feed and water. All groups were immunized twice (on 1 d and 14 d, respectively) with a 2-week interval by intramuscularly inoculated with the volume of 500 μL of vaccine/phosphate-buffered saline (PBS). The chickens in each group were challenged on 28 d with the nvIBDV strains QZ191002 and YL160304 by 10^5^ TCID_50_/0.2 mL per bird via the oral route according to the previously described (20, 50), respectively. The design and vaccination schedule of the experiments are shown in detail in Table 1.

**Table 1:** Design and vaccination schedule of the vaccination-challenge experiments.

Group	Number	First vaccination (1 d)	Secondary vaccination (14 d)	Challenge (28 d)
Vaccine A + YL160304-OEV	10	Vaccine A	YL160304-OEV	QZ191002
Vaccine A + QZ191002-OEV	10	Vaccine A	QZ191002-OEV	
Vaccine B + YL160304-OEV	10	Vaccine B	YL160304-OEV	
Vaccine B + QZ191002-OEV	10	Vaccine B	QZ191002-OEV	
YL160304-OEV + B87	10	YL160304-OEV	B87	
QZ191002-OEV + B87	10	QZ191002-OEV	B87	
NN1172-OEV + B87	10	NN1172-OEV	B87	
YL160304-OEV + YL160304-OEV	10	YL160304-OEV	YL160304-OEV	
QZ191002-OEV + QZ191002-OEV	10	QZ191002-OEV	QZ191002-OEV	
PBS + Vaccine C	10	PBS	Vaccine C	
PBS + PBS	10	PBS	PBS	
Vaccine A + YL160304-OEV	10	Vaccine A	YL160304-OEV	YL160304
Vaccine A + QZ191002-OEV	10	Vaccine A	QZ191002-OEV	
Vaccine B + YL160304-OEV	10	Vaccine B	YL160304-OEV	
Vaccine B + QZ191002-OEV	10	Vaccine B	QZ191002-OEV	
YL160304-OEV + B87	10	YL160304-OEV	B87	
QZ191002-OEV + B87	10	QZ191002-OEV	B87	
NN1172-OEV + B87	10	NN1172-OEV	B87	
YL160304-OEV + YL160304-OEV	10	YL160304-OEV	YL160304-OEV	
QZ191002-OEV + QZ191002-OEV	10	QZ191002-OEV	QZ191002-OEV	
PBS + Vaccine C	10	PBS	Vaccine C	
PBS + PBS	10	PBS	PBS	
Control	10	PBS	PBS	PBS

After the challenge, the clinical signs and possible mortalities were recorded every day. At 7 days post-challenge (dpc), all chickens of each group were euthanized for necropsy. The bursa/body-weight index (BBIX) was calculated to measure the degree of the BF atrophy, and it was considered severe atrophy if BBIX<0.70 (51). Each bursa was divided into two parts for the following analysis: A part of the bursa samples was performed to analyze the viral load by quantitative RT-PCR (52), and the other part was fixed in 4% neutral buffered formalin and stained with HE for further histopathological examination. The results were quantified according to Skeeles’ scale for histopathological bursal lesion scores (HBLSs) (53).

### 2.5 Detection of IBDV-specific antibodies

Serum samples were collected from the wing veins of the immunized and unimmunized chickens at 1, 7, 14, 21, and 28 d, respectively, and the titers of IBDV-specific antibodies were determined by enzyme-linked immunosorbent assay (ELISA) (54). In brief, the purified VP2 protein was diluted with a coating buffer (1.59 g Na_2_CO_3_, 2.93 g NaHCO_3_, 1,000 mL ddH_2_O, pH 9.6) to a final concentration of 0.8 μg/mL. Each well of the ELISA plate was coated with 400 μL of the diluted VP2 protein and incubated at 37°C for 2 h. After washing with PBST buffer (PBS containing 0.05% Tween-20, pH 7.4) for three times to remove unbound antigens and impurities, the wells were blocked with 5% skim milk (BD Difco^™^, United States) at 37°C for 2 h and wash three times as above. Subsequently, 100 μL of chicken serum were added to each well at a 1:100 dilution and incubated at 37°C for 90 min. After washing, 100 μL Rabbit Anti-Chicken IgG Antibody (H + L)-HRP Conjugated (Biosynthesis Biotechnology Inc. Beijing, China), diluted 1:4000, was added and incubated for 30 min. Following another wash, the 100 μL volume of 3,3′,5,5′-tetramethylbenzidine (TMB) ELISA substrate solution (TransGen Biotech, Beijing, China) was added to visualize the reaction and incubated at 37°C for 10 min in the dark. Finally, the reaction was terminated by adding 50 μL of 2 M H_2_SO_4_ to each well, and absorbance values at 450 nm were measured using a Microplate Spectrophotometer.

### 2.6 Flow cytometry analysis of the lymphocytes of CD4^+^, CD8^+^ T, and B

Peripheral blood lymphocytes (PBLs) were isolated from the anticoagulation-treated (with sodium citrate) blood samples that were sampled from the wing veins of the immunized and unimmunized chickens at 1, 7, 14, 21, and 28 d, respectively, as the previously described method (55). In brief, the anticoagulation-treated blood samples were gently added above the liquid level of the peripheral blood lymphocyte isolation solution, followed by centrifugation at 800 × *g* for 20 min to collect the PBLs. PBLs were stained with Mouse Anti-Chicken CD4-APC, Mouse Anti-Chicken CD8a-FITC, and Mouse Anti-Chicken Bu-1-PE antibodies (SouthernBiotech, Birmingham, United States) according to the manufacturer’s instructions. Finally, the percentages of CD4^+^, CD8^+^ T lymphocytes, and B lymphocyte in all samples were evaluated by Invitrogen Attune™ NxT Flow Cytometer.

### 2.7 Statistical analysis

All data analyses were processed with GraphPad Prism 8.0 software and expressed as means ± standard deviation (SD). Statistical differences among the groups were evaluated using one-way analysis of variance (ANOVA). To compare the differences among treatments, Tukey’s multiple comparisons test was used. The results with *p*-values of <0.05 *, *p* < 0.01 **, *p* < 0.001 ***, and *p* < 0.0001 **** were considered statistically significant.

## 3 Results

### 3.1 The anti-IBDV antibody detection and B lymphocyte count revealed a sustained high level of humoral immune response in the immunized chickens

**Figure 1:**
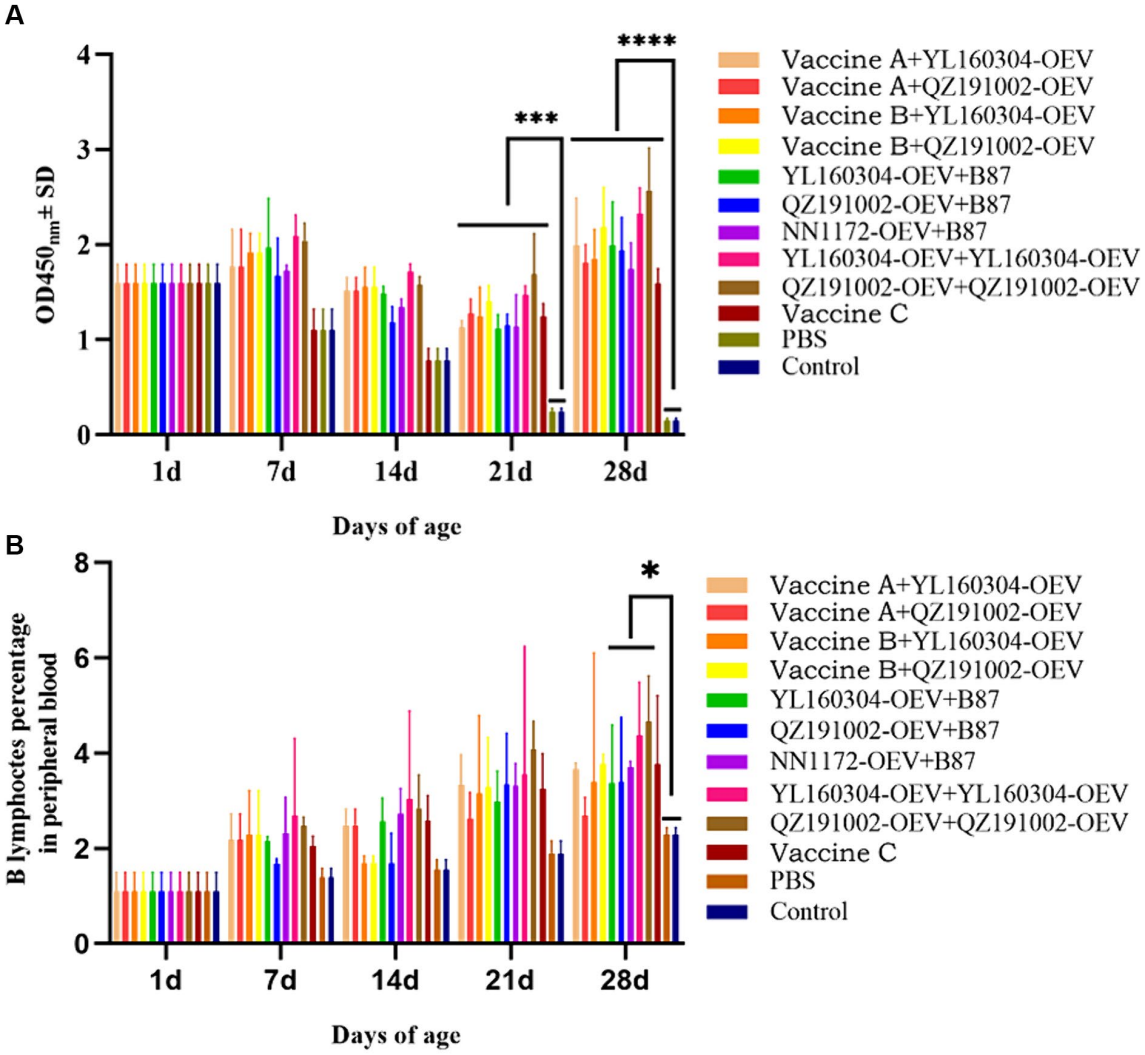
Anti-IBDV antibody detection and the percentages of B lymphocyte in PBLs of the experiment groups. **(A)** The anti-IBDV antibody levels at 1, 7, 14, 21, and 28 d, respectively, detected by ELISA. **(B)** The percentages of B lymphocyte in PBLs, which were isolated from the immunized chickens at 1, 7, 14, 21, and 28 d, respectively, analyzed by flow cytometry.

The antibody responses of chickens induced by different vaccines were detected by the anti-IBDV antibody and B lymphocyte count. Chickens vaccinated with nvIBDV OEVs and the commercial vaccines all produced different levels of antibodies (Figure 1A). The antibody titer of the unimmunized chickens decreased after hatching and reached levels of lower on 21 d and the lowest on 28 d. While those of the immunized chickens gradually increased over time after a short decline period and reached the highest level on the 28 d, indicating that all immunized chickens showed clear serological conversion (acquired immunity). In addition, the antibody levels of the groups that immunized twice with the nvIBDV OEVs were all the highest levels during the experiment, and the groups that with vaccine B (the immunogenic complex vaccine) as the prime and with nvIBDV OEVs as the booster immunizations were the second high. The number of B lymphocyte in all vaccinated groups continued to increase over time after immunization, significantly higher than those in the unimmunized groups (Figure 1B). It is worth noting that the anti-IBDV antibody level and the number of B lymphocyte of the nvIBDV OEV-immunized groups were the highest in all the immunized groups, and its stimulating effect on the humoral immunity seems to be stronger than the current commercial vaccines.

### 3.2 IBDV-specific T-cell immune responses in the immunized chickens

**Figure 2:**
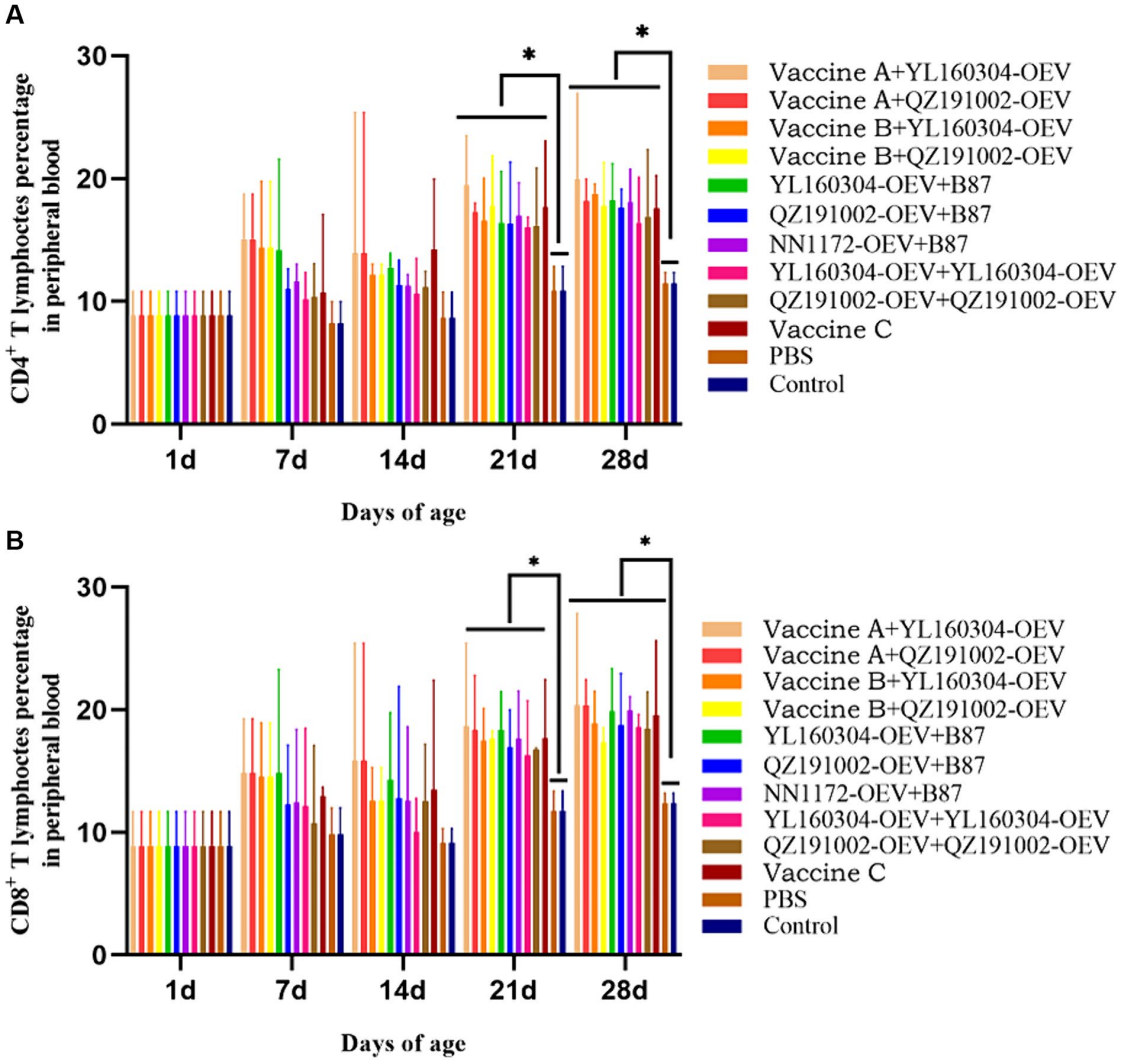
Percentages of CD4^+^ and CD8^+^ T lymphocytes in PBLs of the experiment groups. PBLs were isolated from immunized chickens at 0, 7, 14, 21, and 28 d, respectively, and analyzed by flow cytometry. **(A)** The percentages of CD4^+^ T lymphocytes. **(B)** The percentages of CD8^+^ T lymphocytes.

The T-cell immune response level of the immunized chickens was detected by the percentages of CD4^+^ and CD8^+^ T lymphocytes in the PBLs by flow cytometry (Figure 2). The results showed that the CD4^+^ and CD8^+^ T lymphocytes in all the immunized groups were increased in varying degrees from 7 to 28 d. On 21 d and 28 d, the values in each immunized group were significantly higher than those in the unimmunized group (*p* < 0.05). Most notably, the values of the nvIBDV OEV groups were increased relatively slower in the early stage (7–14 d) but reached a higher level later on 21 d and 28 d.

### 3.3 The booster immunization with different current commercial vaccines still cannot provide complete protection against the nvIBDV

**Figure 3:**
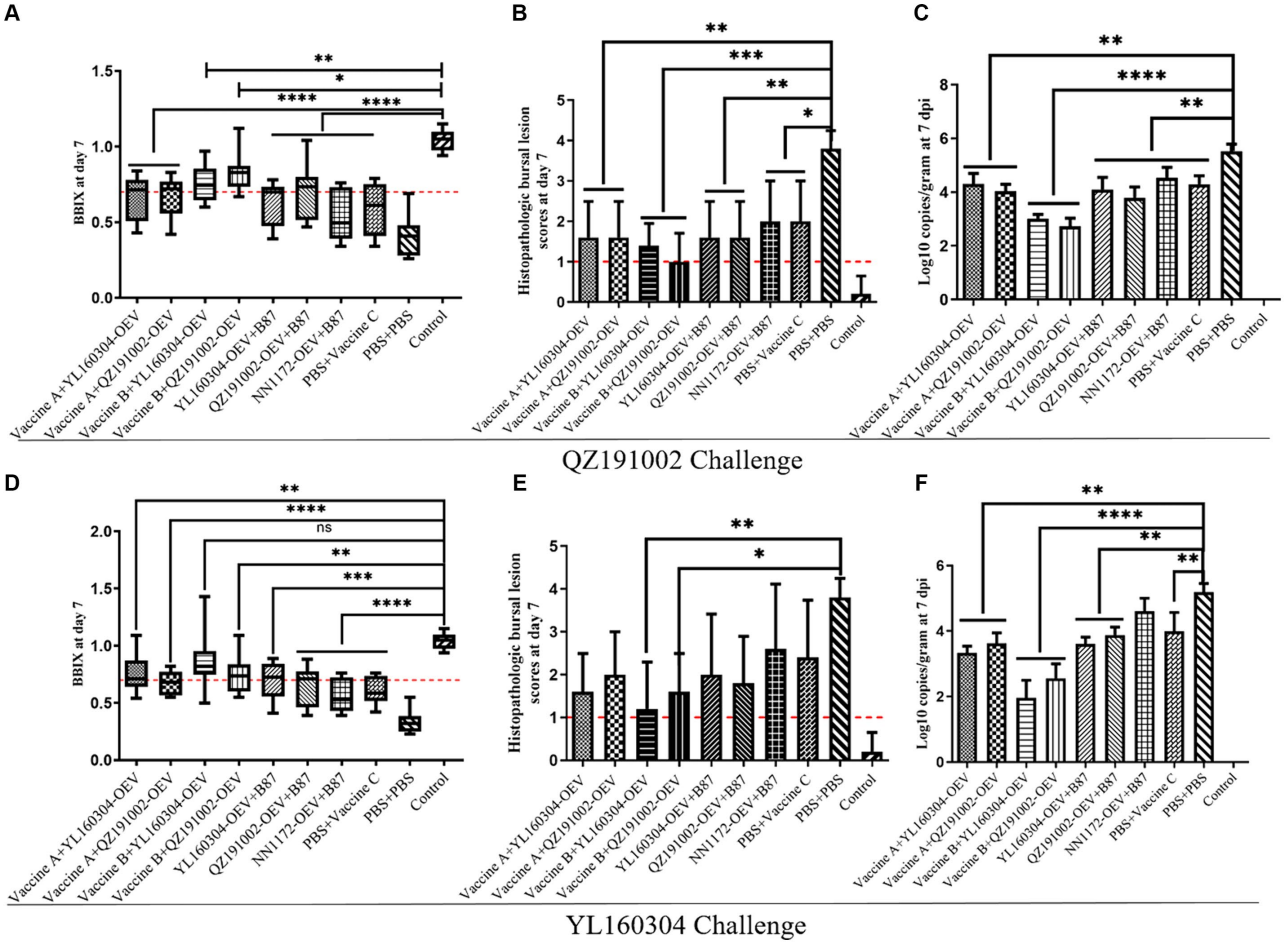
BBIX, HBLS, and viral loads of the groups with booster immunization with the different vaccines and challenged with the nvIBDV strains. **(A–C)** The BBIX, HBLS, and viral loads of the BF, respectively, in the booster immunization groups challenged with QZ191002. **(D–F)** The BBIX, HBLS, and viral loads, respectively, in the booster immunization groups challenged with YL160304.

Under the challenges of nvIBDV strain QZ191002 and its reassortant strain YL160304, no obvious clinical symptoms and death were observed in the chickens immunized with the commercial vaccines and the nvIBDV OEVs (Figure 3). Compared with the control group, severe atrophy of BF was observed in the unimmunized challenged group (BBIX<0.5). The booster immunization of nvIBDV OEVs based on the prime immunization with commercial vaccine B showed relatively mild bursal atrophy when compared to other groups that only immunized with the commercial vaccines (BBIX>0.7) (Figures 3A,D). Similarly, the same phenomenon can be observed in terms of HBLS (Figures 3B,E) and viral load (Figures 3C,F). All data indicated that the booster immunization of the commercial vaccines currently used still cannot provide complete protection against the nvIBDV. However, the booster immunization with the nvIBDV OEVs on the prime immunization with the commercial vaccine B seemed to have a better protection effect than others.

### 3.4 The nvIBDV OEVs can provide complete protection against the homologous virus and YL160304-OEV performs better than QZ191002-OEV

**Figure 4:**
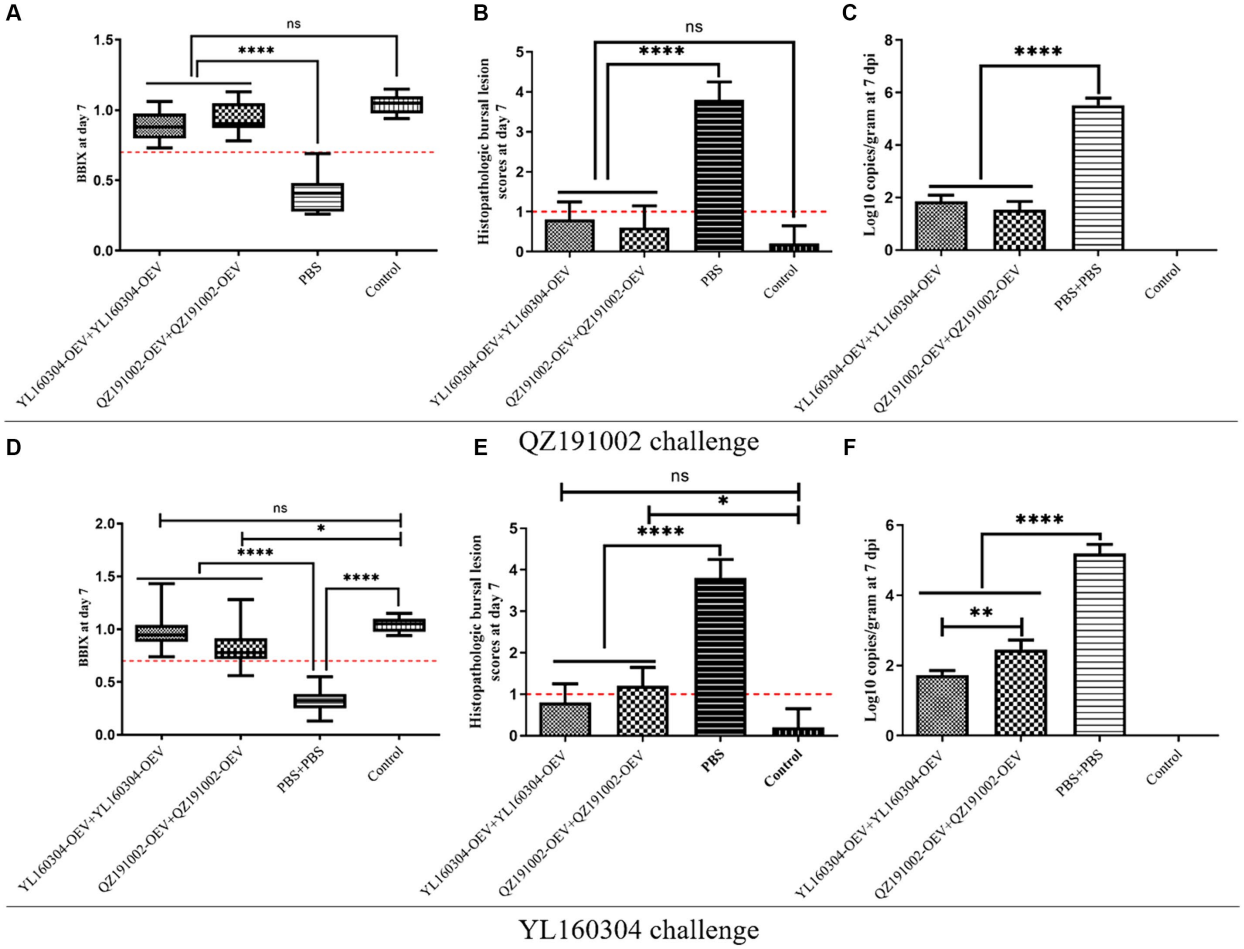
BBIX, HBLS, and viral loads of the nvIBDV OEV-immunized groups challenged with the nvIBDV strains. **(A–C)**The BBIX, HBLS, and viral loads, respectively, in the nvIBDV OEV-immunized groups challenged with QZ191002 strain. **(D–F)** The BBIX, HBLS, and viral loads, respectively, of the nvIBDV OEV-immunized groups challenged with YL160304 strain.

In the groups vaccinated with nvIBDV OEVs, no significant morbidity or mortality was observed in chickens challenged with QZ191002 or YL160304, as in the control group (Figure 4). The unimmunized group challenged with YL160304 strain resulted in 10% (1/10) of the mortality rate, while the unimmunized group challenged with QZ191002 showed no obvious clinical symptoms or mortality. Further study on the values of BBIX, HBLS, and viral load showed that no significant bursal atrophy (BBIX>0.7) (Figures 4A,D) and histopathologic bursal lesions (HBLS<1) (Figures 4B,E) were observed in the nvIBDV OEV-immunized group. However, significant atrophy (BBIX<0.5) and severe histopathologic lesions in the BF (HBLS>3) can be observed in the unimmunized challenged group. The viral loads in the immunized groups were significantly lower than that in the unimmunized challenged group, which is consistent with the phenomenon observed by HBLS (Figures 4C,F). Interestingly, the data also revealed that the degree of damage to the BF is related to the replication efficiency of the virus (viral load) in the organ. It is worth noting that the YL160304-OEV (A-nv/B-HLJ0504-like) showed to be able to provide better protection against the challenge than those of the QZ191002-OEV (A-nv/B-nv), indicating that VP1 of IBDV is not only involved in the virulence of the virus but also has a certain impact on the antigenicity of the virus (Figures 4D–F).

### 3.5 Protective efficiency of the current commercial vaccines and the nvIBDV OEVs against the nvIBDVs challenge

The protective efficiency of nvIBDV OEVs and different commercial vaccines in booster immunization against the challenge of nvIBDVs were further clarified according to the BBIX, HBLS, and viral loads of the BF from the immunization-challenge experiments, as shown in Table 2. The results showed that nvIBDV could break through the protections provided by only one immunization dose of the current commercial vaccines, with the protection rate usually being 40%–60%. Interestingly, even with booster immunization with different current commercial vaccines, the protection rates could only be increased to 60%–80%, indicating complete protection could not be provided. As expected, only the homologous nvIBDV OEVs could provide 100% protections against the challenges of the homologous strains, which could induce high-level specific antibodies, ameliorate target organ damage, and significantly reduce viral load (Figure 3). Notably, YL160304-OEV performed better than QZ191002-OEV, providing 100% protection not only against the challenge of homologous strain but also against that of heterologous QZ191002 strain (Table 2).

**Table 2:** Protective efficiency of the current commercial vaccines and nvIBDV OEVs in the immunized groups challenged with the nvIBDV strains.

Group	Challenge	Viral load in BF (Log^10^ copies/g)	Bursa/body-weight index (BBIX) ratio											Histopathological bursal lesion scores					
			1	2	3	4	5	6	7	8	9	10	Protection	0	1	2	3	4	Protection
Vaccine A + YL160304-OEV	QZ191002	4.31	+	+	–	–	+	+	–	–	+	+	60%	0	3	1	1	0	60%
Vaccine A + QZ191002-OEV		4.05	+	–	+	+	–	+	+	+	–	–	60%	0	3	1	1	0	60%
Vaccine B + YL160304-OEV		3.75	+	+	+	–	+	–	+	–	+	+	70%	0	3	2	0	0	60%
Vaccine B + QZ191002-OEV		2.73	+	+	+	+	+	+	+	+	–	+	90%	1	3	1	0	0	80%
YL160304-OEV + B87		4.1	–	–	+	–	+	+	–	+	+	+	60%	0	3	1	1	0	60%
QZ191002-OEV + B87		3.79	+	+	–	+	+	–	+	–	+	+	70%	0	3	1	1	0	60%
NN1172-OEV + B87		4.54	+	+	–	–	–	–	–	–	+	+	40%	0	2	1	2	0	40%
YL160304-OEV + YL160304-OEV		2.82	+	+	+	+	+	+	+	+	–	+	90%	1	4	0	0	0	100%
QZ191002-OEV + QZ191002-OEV		1.96	+	+	+	+	+	+	+	+	+	+	100%	2	3	0	0	0	100%
PBS + Vaccine C		4.29	–	+	+	+	–	+	–	+	–	–	50%	0	2	1	2	0	40%
PBS + PBS		5.52	–	–	–	–	–	–	–	–	–	–	0%	0	0	0	1	4	0%
Vaccine A + YL160304-OEV	YL160304	3.33	–	+	–	+	+	+	+	+	+	–	70%	0	3	1	1	0	60%
Vaccine A + QZ191002-OEV		3.63	–	+	+	–	–	+	–	+	+	–	50%	0	2	1	2	0	40%
Vaccine B + YL160304-OEV		2.5	+	+	+	+	+	+	+	–	+	+	90%	1	3	0	1	0	80%
Vaccine B + QZ191002-OEV		3.89	–	+	–	+	+	+	+	–	+	–	60%	0	3	0	2	0	60%
YL160304-OEV + B87		3.6	+	+	+	–	+	+	–	+	+	–	70%	0	3	0	1	1	60%
QZ191002-OEV + B87		3.88	+	+	+	–	+	–	–	+	+	–	60%	0	3	0	2	0	60%
NN1172-OEV + B87		4.6	–	+	–	+	–	–	+	+	–	–	40%	0	2	0	1	2	40%
YL160304-OEV + YL160304-OEV		1.73	+	+	+	+	+	+	+	+	+	+	100%	1	4	0	0	0	100%
QZ191002-OEV + QZ191002-OEV		2.45	–	+	+	+	+	+	+	+	+	+	90%	0	4	1	0	0	80%
PBS + Vaccine C		3.99	+	+	–	–	–	–	+	–	–	+	40%	0	2	0	2	1	40%
PBS + PBS		5.19	–	–	–	–	–	–	–	–	–	–	0%	0	0	0	0	5	0%
Control		0	+	+	+	+	+	+	+	+	+	+	–	4	1	0	0	0	–

## 4 Discussion

Although a large-scale immunization program against IBDV infection has been carried out in the poultry industry, more and more different types of IBDV were isolated increasingly year by year from the vaccinated chickens (56). At present, many IBDVs with different path−/geno−/serotypes have been confirmed to be co-circulating in China, and the current commercial vaccines cannot provide sufficient protection, especially for the newly emerging nvIBDV (20–22, 45, 57, 58). It is reported that the epidemic of nvIBDV occurred in the important poultry-raising areas of China during 2016–2022, and the infection and prevalence of the nvIBDV strain were found in 17 provinces, including the major chicken production provinces like Shandong, Guangxi, and Hebei (17, 21, 57, 59–61). Due to the frequent commercial trade of live poultry and products, nvIBDV has spread to Southern China, Eastern China, and Northeast China, with Central China as the major transmission source, and it has even emerged and spread rapidly in immunized chickens in East Asia (Japan and South Korea) and Southeast Asia (Malaysia) (21), which seems to have a trend of further spreading to other regions of the world. To date, the raised calls for frequent outbreaks of nvIBDV among the vaccinated chickens in Asian countries/regions have raised the significance of thinking about vaccine effectiveness.

IBDV belongs to the double-stranded RNA viruses, and its conformation makes it more prone to variation. Previous studies have indicated that IBDV mainly evolves in diversity through gene mutation and reassortment to escape the host’s immune mechanisms and facilitate better survival of its own virus (20, 21, 27, 30, 62–64). Although China has made efforts to control the disease through regular immunization with vaccines against vvIBDV, the emergence of nvIBDV is raising new challenges. In this study, the twice immunization of current commercial vaccines (subunit vaccine, killed vaccine, or intermediate-plus live vaccine all mainly targeting the epidemic vvIBDV strains) has failed to provide complete protection against the nvIBDV (60%–80%), although they can provide better protection than previously reported once immunization (40%–60%) (44, 45). Leng et al. (65) introduced the “placeholder effect” as a novel criterion for assessing vaccine effectiveness, showing that the W2512 vaccine causes severe atrophy of the BF in SPF chickens and Yellow chickens, induces high levels of antibodies against IBDV, and protects chickens from infection with the novel variant strains via a placeholder effect. The results of this study indicate that developing an efficient vaccine against the increasing prevalence of nvIBDV is crucial for the prevention and control of the disease right now and in future.

Essentially, the difference in protection rates is probably related to the variation of VP2 between the field and vaccine strains. VP2 is considered as a crucial structural protein of IBDV and an important dominant antigen for vaccine development. However, many researchers have confirmed that the change of some specific amino acids in the VP2 region might lead to virus antigen drift, further reducing the ability of antibodies to capture the virus (57, 66–68). Compared to vvIBDV and attIBDV strains, changes in multiple antigenic sites have been found in nvIBDV (21, 57). This high mutation rate helps to enable it to escape the capture of the immune system induced by the current commercial vaccines and persist for long periods in the immunopotent chickens. As found in this study, twice immunizations (1, 14 d) of nvIBDV OEVs can provide good protection (90%–100%) against the epidemic strains of nvIBDV, which is superior to the protection (60%–80%) provided by the traditional commercial vaccines’ immunization (primary immunization) and nvIBDV (boost immunization) in the experiment. It suggests that to provide complete protection against nvIBDV strains, boost immunization with nvIBDV OEVs may be necessary. This poses new challenges to the IBD vaccination programs of regions or countries that are affected by the emergence of nvIBDV in current or in future. As in addition to the emerging nvIBDV, there are also co-circulations of vvIBDV (which are still dominant strains in many places) and/or cIBDV in the field. So, developing vaccines of multi-viral antigens that include multiple types of viruses is a possible solution.

More and more studies indicate that VP1 of IBDV is not only involved in the virulence of the virus (25, 26, 29, 32, 69, 70) but also seems to have a certain impact on the antigenicity of the virus. Chen et al. found that NN1005 (A-vv/B-new type) and JS7 (A-vv/B-c), two OEVs with the same VP2, exhibit different protection rates due to their different origins of VP1 (49). This has also been confirmed in this immunization-challenge experiment; the comparative study of two different genotypes of nvIBDV showed that the protective spectrum of YL160304-OEV (A-nv/B-HLJ0504-like vvIBDV) was superior to QZ191002-OEV (A-nv/B-nv), indicating that YL160304 strain could be a good candidate for the development of new vaccine against the nvIBDVs. Through such reassortants (VP1 originating from vvIBDV), the YL160304 strain might enhance its pathogenicity to chickens by increasing the virulence of the virus (20) and might also escape from the body’s immunity by altering the viral antigenicity, thereby increasing the chances of its survival and epidemic spread in the vaccinated chickens (49). Furthermore, the same results were also confirmed in the role of VP1 in the pathogenicity of the virus strain in this challenge experiment, which showed that the pathogenicity of YL160304 strain (10% mortality) with its VP1 originated from HLJ0504-like to three-yellow chicken was significantly higher than that of QZ191002 (0% mortality), indicating that VP1 reassortment contributes to enhance the virulence of nvIBDV. Therefore, in addition to be recognized and proven as the most important protective antigen of VP2, different origins of VP1 might also play an important role in the antigenicity of IBDV, although this still needs to be further verified by the construct of recombinant virus with different origins of VP2 and VP1 by the reverse genetics.

## 5 Conclusion

At present, many IBDVs with different path−/geno−/serotypes have been confirmed to be co-circulating in China and some other Asian countries, and the current commercial vaccines cannot provide sufficient protection, especially against the newly emerging nvIBDV strains. Our results suggest that even the booster immunization with the current commercial vaccines still cannot provide complete protection against the nvIBDVs, while the nvIBDV OEVs can provide complete protection. In addition, YL160304-OEV (A-nv/B-HLJ0504-like) can provide a broader protective spectrum against different nvIBDV strains. The findings of this study provide important insights in the current vaccination practice procedures of the nvIBDV and will assist in the development of programs for control strategies for these emerging viruses.

## Data Availability

The original contributions presented in the study are included in the article/supplementary material, further inquiries can be directed to the corresponding authors.
